# 2,3-Diamino­pyridinium benzoate

**DOI:** 10.1107/S1600536809021011

**Published:** 2009-06-06

**Authors:** Kasthuri Balasubramani, Hoong-Kun Fun

**Affiliations:** a X-ray Crystallography Unit, School of Physics, Universiti Sains Malaysia, 11800 USM, Penang, Malaysia

## Abstract

In the title compound, C_5_H_8_N_3_^+^·C_7_H_5_O_2_^−^, the pyridine N atom is protonated. The carboxyl­ate group of the benzoate anion is twisted away from the attached ring by 10.91 (9)°. In the crystal structure, N—H⋯O hydrogen bonds between 2,3-diamino­pyridinium cations and benzoate anions, and π–π inter­actions between the pyridinium rings [centroid–centroid distance = 3.6467 (9) Å] form a two-dimensional network parallel to (001). In the network, N—H⋯O hydrogen bonds form *R*_2_^2^(8) and *R*_2_^1^(7) ring motifs.

## Related literature

For general background to pyridine derivatives, see: Pozharski *et al.* (1997 ▶); Katritzky *et al.* (1996 ▶). For bond-length data, see: Allen *et al.* (1987 ▶). For details of hydrogen bonding, see: Jeffrey & Saenger (1991 ▶); Jeffrey (1997 ▶); Scheiner (1997 ▶). For hydrogen-bond motifs, see: Bernstein *et al.* (1995 ▶). For the stability of the temperature controller used in the data collection, see: Cosier & Glazer (1986 ▶).
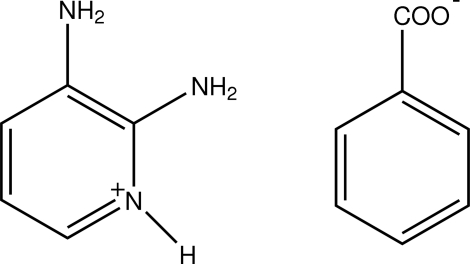


## Experimental

### 

#### Crystal data

C_5_H_8_N_3_^+^·C_7_H_5_O_2_^−^
*M* *_r_* = 231.25
Orthorhombic, 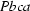

*a* = 10.1498 (3) Å
*b* = 11.0656 (3) Å
*c* = 20.7368 (7) Å
*V* = 2329.03 (12) Å^3^
*Z* = 8
Mo *K*α radiation
μ = 0.09 mm^−1^
*T* = 100 K
0.43 × 0.40 × 0.03 mm

#### Data collection

Bruker SMART APEXII CCD area-detector diffractometer
Absorption correction: multi-scan (*SADABS*; Bruker, 2005 ▶) *T*_min_ = 0.935, *T*_max_ = 0.998
27109 measured reflections
3443 independent reflections
2559 reflections with *I* > 2σ(*I*)
*R*_int_ = 0.070

#### Refinement

*R*[*F* ^2^ > 2σ(*F* ^2^)] = 0.065
*wR*(*F* ^2^) = 0.124
*S* = 1.09
3443 reflections
206 parameters
H atoms treated by a mixture of independent and constrained refinement
Δρ_max_ = 0.26 e Å^−3^
Δρ_min_ = −0.22 e Å^−3^

### 

Data collection: *APEX2* (Bruker, 2005 ▶); cell refinement: *SAINT* (Bruker, 2005 ▶); data reduction: *SAINT*; program(s) used to solve structure: *SHELXTL* (Sheldrick, 2008 ▶); program(s) used to refine structure: *SHELXTL*; molecular graphics: *SHELXTL*; software used to prepare material for publication: *SHELXTL* and *PLATON* (Spek, 2009 ▶).

## Supplementary Material

Crystal structure: contains datablocks global, I. DOI: 10.1107/S1600536809021011/ci2821sup1.cif

Structure factors: contains datablocks I. DOI: 10.1107/S1600536809021011/ci2821Isup2.hkl

Additional supplementary materials:  crystallographic information; 3D view; checkCIF report

## Figures and Tables

**Table 1:** Hydrogen-bond geometry (Å, °)

*D*—H⋯*A*	*D*—H	H⋯*A*	*D*⋯*A*	*D*—H⋯*A*
N1—H1*N*1⋯O2	0.96 (2)	1.77 (2)	2.7218 (18)	176 (2)
N2—H1*N*2⋯O1	0.90 (2)	1.94 (2)	2.8377 (18)	173 (2)
N2—H2*N*2⋯O2^i^	0.88 (2)	2.01 (2)	2.8873 (17)	170 (2)
N3—H1*N*3⋯O2^i^	0.91 (2)	2.02 (2)	2.9206 (19)	173 (2)
N3—H2*N*3⋯O1^ii^	0.95 (2)	2.00 (2)	2.9382 (19)	170 (2)

Symmetry codes: (i) 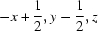; (ii) 
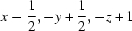
.

**Fig. 1.**
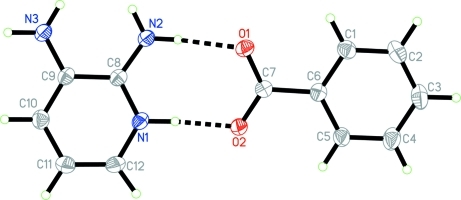
The asymmetric unit of the title compound, showing 50% probability displacement ellipsoids and the atom-numbering scheme. Dashed lines indicate hydrogen bonds.

**Fig. 2.**
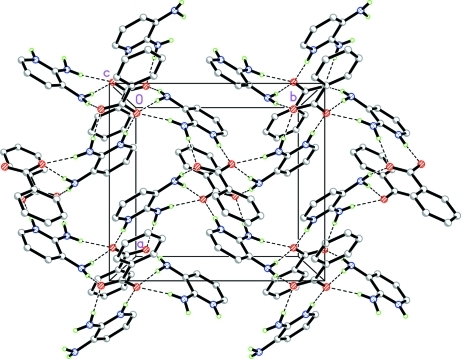
Part of the crystal packing of the title compound. Dashed lines indicate hydrogen bonds.

## supplementary crystallographic information

### Comment

Pyridine and its derivatives play an important role in heterocyclic chemistry (Pozharski *et al.*, 1997; Katritzky *et al.*, 1996). Pyridine and its substituted derivatives are often involved in hydrogen-bond interactions (Jeffrey & Saenger, 1991; Jeffrey, 1997; Scheiner, 1997). Since our aim is to study some interesting hydrogen-bonding interactions, the crystal structure of the title compound is presented here.

The asymmetric unit (Fig 1), contains a protonated 2,3-diaminopyridinium cation and a benzoate anion. The bond lengths (Allen *et al.*,1987) and angles are normal. In the 2,3-diaminopyridinium cation, the protonated N1 atom has lead to a slight increase in C8—N1—C12 angle to 123.30 (14)°. Moreover, the carboxylate group is twisted slightly out of the attached ring; the dihedral angle between C1—C6 and O1/O2/C7/C6 planes is 10.91 (9)°. The 2,3-diaminopyridinium cation is planar, with a maximum deviation of 0.0089 (17) Å for atom C9.

In the crystal packing, the protonated N1 atom and the 2-amino group (N2) is hydrogen-bonded to the carboxylate oxygen atoms (O1 and O2) *via* a pair of N—H···O hydrogen bonds forming an *R*_2_^2^(8) ring motif (Bernstein *et al.*, 1995). The two amino groups (N2 and N3) are involved in N—H···O hydrogen bonding interactions to form an *R*^1^_2_(7) ring motif. The cationic and anionic units are linked through N—H···O hydrogen bonds (Table 1 and Fig 2) to form a two-dimensional network parallel to the (001) plane. The crystal structure is further stabilized by π-π stacking interactions between the pyridinium rings of the cations at (x, y, z) and (-x, 1-y, 1-z), with a centroid to centroid distance of 3.6467 (9) Å.

### Experimental

Hot methanol solutions (20 ml) of 2,3-diaminopyridine (27 mg, Aldrich) and benzoic acid (31 mg, Merck) were mixed and warmed over a heating magnetic stirrer for 5 minutes. The resulting solution was allowed to cool slowly at room temperature. Crystals of the title compound appeared from the mother liquor after a few days.

### Refinement

All H atoms were located in a difference Fourier map and allowed to refine freely [N-H = 0.89 (2)–0.95 (2) Å and C–H = 0.97 (18)–1.02 (2) Å].

### Crystal data

**Table:**

C_5_H_8_N_3_^+^·C_7_H_5_O_2_^−^	*F*(000) = 976
*M**_r_* = 231.25	*D*_x_ = 1.319 Mg m^−^^3^
Orthorhombic, *P**b**c**a*	Mo *K*α radiation, λ = 0.71073 Å
Hall symbol: -P 2ac 2ab	Cell parameters from 3782 reflections
*a* = 10.1498 (3) Å	θ = 2.8–27.9°
*b* = 11.0656 (3) Å	µ = 0.09 mm^−^^1^
*c* = 20.7368 (7) Å	*T* = 100 K
*V* = 2329.03 (12) Å^3^	Plate, brown
*Z* = 8	0.43 × 0.40 × 0.03 mm

### Data collection

**Table:**

Bruker SMART APEXII CCD area-detector diffractometer	3443 independent reflections
Radiation source: fine-focus sealed tube	2559 reflections with *I* > 2σ(*I*)
graphite	*R*_int_ = 0.070
φ and ω scans	θ_max_ = 30.1°, θ_min_ = 2.9°
Absorption correction: multi-scan (*SADABS*; Bruker, 2005)	*h* = −14→14
*T*_min_ = 0.935, *T*_max_ = 0.998	*k* = −15→15
27109 measured reflections	*l* = −26→29

### Refinement

**Table:**

Refinement on *F*^2^	Primary atom site location: structure-invariant direct methods
Least-squares matrix: full	Secondary atom site location: difference Fourier map
*R*[*F*^2^ > 2σ(*F*^2^)] = 0.065	Hydrogen site location: inferred from neighbouring sites
*wR*(*F*^2^) = 0.124	H atoms treated by a mixture of independent and constrained refinement
*S* = 1.09	*w* = 1/[σ^2^(*F*_o_^2^) + (0.0394*P*)^2^ + 1.0527*P*] where *P* = (*F*_o_^2^ + 2*F*_c_^2^)/3
3443 reflections	(Δ/σ)_max_ = 0.001
206 parameters	Δρ_max_ = 0.26 e Å^−^^3^
0 restraints	Δρ_min_ = −0.22 e Å^−^^3^

### Special details

**Table:**

Experimental. The crystal was placed in the cold stream of an Oxford Cyrosystems Cobra open-flow nitrogen cryostat (Cosier & Glazer, 1986) operating at 100.0 (1) K.
Geometry. All e.s.d.'s (except the e.s.d. in the dihedral angle between two l.s. planes) are estimated using the full covariance matrix. The cell e.s.d.'s are taken into account individually in the estimation of e.s.d.'s in distances, angles and torsion angles; correlations between e.s.d.'s in cell parameters are only used when they are defined by crystal symmetry. An approximate (isotropic) treatment of cell e.s.d.'s is used for estimating e.s.d.'s involving l.s. planes.
Refinement. Refinement of F^2^ against ALL reflections. The weighted R-factor wR and goodness of fit S are based on F^2^, conventional R-factors R are based on F, with F set to zero for negative F^2^. The threshold expression of F^2^ > σ(F^2^) is used only for calculating R-factors(gt) etc. and is not relevant to the choice of reflections for refinement. R-factors based on F^2^ are statistically about twice as large as those based on F, and R- factors based on ALL data will be even larger.

### Fractional atomic coordinates and isotropic or equivalent isotropic displacement parameters (Å^2^)

**Table:**

	*x*	*y*	*z*	*U*_iso_*/*U*_eq_	
O1	0.41320 (11)	0.39789 (9)	0.63510 (5)	0.0253 (3)	
O2	0.39681 (11)	0.57595 (9)	0.58541 (5)	0.0257 (3)	
C6	0.54129 (15)	0.55976 (13)	0.67572 (8)	0.0217 (3)	
C1	0.57778 (16)	0.49395 (15)	0.73019 (8)	0.0271 (4)	
C2	0.66687 (18)	0.54115 (17)	0.77390 (10)	0.0347 (4)	
C3	0.72160 (19)	0.65474 (16)	0.76378 (10)	0.0365 (4)	
C4	0.68658 (19)	0.72004 (16)	0.70966 (10)	0.0375 (5)	
C5	0.59687 (17)	0.67368 (14)	0.66578 (9)	0.0296 (4)	
C7	0.44423 (15)	0.50698 (13)	0.62917 (7)	0.0211 (3)	
C9	0.06661 (15)	0.32936 (13)	0.46855 (8)	0.0220 (3)	
C8	0.15562 (15)	0.37759 (13)	0.51515 (8)	0.0211 (3)	
N1	0.22115 (13)	0.47997 (11)	0.50049 (7)	0.0230 (3)	
N2	0.17608 (14)	0.32713 (12)	0.57320 (7)	0.0239 (3)	
N3	−0.00614 (14)	0.22766 (12)	0.48334 (8)	0.0273 (3)	
C12	0.20715 (17)	0.53936 (14)	0.44310 (9)	0.0270 (4)	
C11	0.12491 (17)	0.49519 (15)	0.39762 (9)	0.0288 (4)	
C10	0.05443 (17)	0.38821 (14)	0.41056 (9)	0.0268 (4)	
H12A	0.262 (2)	0.6122 (17)	0.4384 (9)	0.039 (5)*	
H11A	0.1152 (17)	0.5333 (16)	0.3555 (9)	0.028 (5)*	
H10A	−0.0033 (18)	0.3556 (15)	0.3778 (9)	0.027 (5)*	
H1A	0.5401 (19)	0.4138 (17)	0.7363 (9)	0.037 (5)*	
H2A	0.690 (2)	0.4955 (18)	0.8134 (10)	0.045 (6)*	
H3A	0.785 (2)	0.6890 (17)	0.7969 (10)	0.043 (6)*	
H4A	0.726 (2)	0.7970 (19)	0.7013 (10)	0.051 (6)*	
H5A	0.5737 (18)	0.7200 (17)	0.6276 (9)	0.032 (5)*	
H1N1	0.283 (2)	0.5096 (18)	0.5312 (9)	0.039 (5)*	
H1N2	0.248 (2)	0.3487 (16)	0.5959 (9)	0.033 (5)*	
H2N2	0.146 (2)	0.253 (2)	0.5800 (10)	0.047 (6)*	
H1N3	0.021 (2)	0.1812 (18)	0.5170 (10)	0.042 (6)*	
H2N3	−0.043 (2)	0.1882 (17)	0.4469 (10)	0.041 (6)*	

### Atomic displacement parameters (Å^2^)

**Table:**

	*U*^11^	*U*^22^	*U*^33^	*U*^12^	*U*^13^	*U*^23^
O1	0.0278 (6)	0.0181 (5)	0.0298 (6)	−0.0009 (4)	−0.0021 (5)	0.0002 (4)
O2	0.0280 (6)	0.0205 (5)	0.0285 (6)	−0.0020 (4)	−0.0058 (5)	0.0020 (4)
C6	0.0181 (7)	0.0209 (7)	0.0261 (8)	0.0037 (5)	0.0001 (6)	−0.0025 (6)
C1	0.0252 (8)	0.0271 (8)	0.0291 (9)	0.0039 (6)	−0.0009 (7)	0.0015 (7)
C2	0.0335 (10)	0.0398 (10)	0.0308 (10)	0.0067 (8)	−0.0089 (8)	0.0007 (8)
C3	0.0339 (10)	0.0346 (9)	0.0410 (11)	0.0058 (7)	−0.0150 (8)	−0.0104 (8)
C4	0.0361 (10)	0.0245 (8)	0.0520 (12)	−0.0009 (7)	−0.0165 (9)	−0.0026 (8)
C5	0.0298 (9)	0.0227 (7)	0.0364 (10)	0.0003 (6)	−0.0111 (8)	0.0019 (7)
C7	0.0197 (7)	0.0210 (7)	0.0226 (8)	0.0019 (5)	0.0027 (6)	−0.0013 (6)
C9	0.0194 (7)	0.0182 (7)	0.0285 (9)	0.0017 (5)	−0.0004 (6)	−0.0027 (6)
C8	0.0192 (7)	0.0175 (6)	0.0265 (8)	0.0028 (5)	0.0011 (6)	−0.0016 (6)
N1	0.0223 (7)	0.0186 (6)	0.0281 (7)	−0.0011 (5)	−0.0013 (6)	−0.0012 (5)
N2	0.0237 (7)	0.0213 (6)	0.0269 (8)	−0.0029 (5)	−0.0020 (6)	0.0014 (5)
N3	0.0288 (7)	0.0217 (6)	0.0314 (8)	−0.0048 (5)	−0.0055 (7)	0.0010 (6)
C12	0.0286 (9)	0.0204 (7)	0.0320 (9)	−0.0014 (6)	0.0008 (7)	0.0038 (6)
C11	0.0319 (9)	0.0260 (8)	0.0285 (9)	0.0006 (7)	−0.0019 (7)	0.0054 (7)
C10	0.0252 (8)	0.0269 (8)	0.0283 (9)	0.0000 (6)	−0.0043 (7)	−0.0014 (7)

### Geometric parameters (Å, °)

**Table:**

O1—C7	1.2537 (17)	C9—N3	1.3805 (19)
O2—C7	1.2796 (18)	C9—C8	1.427 (2)
C6—C1	1.394 (2)	C8—N2	1.343 (2)
C6—C5	1.396 (2)	C8—N1	1.3484 (19)
C6—C7	1.498 (2)	N1—C12	1.367 (2)
C1—C2	1.383 (2)	N1—H1N1	0.96 (2)
C1—H1A	0.974 (19)	N2—H1N2	0.90 (2)
C2—C3	1.390 (3)	N2—H2N2	0.88 (2)
C2—H2A	0.99 (2)	N3—H1N3	0.91 (2)
C3—C4	1.381 (3)	N3—H2N3	0.95 (2)
C3—H3A	1.02 (2)	C12—C11	1.351 (2)
C4—C5	1.386 (2)	C12—H12A	0.99 (2)
C4—H4A	0.96 (2)	C11—C10	1.409 (2)
C5—H5A	0.972 (19)	C11—H11A	0.975 (18)
C9—C10	1.373 (2)	C10—H10A	0.966 (18)
			
C1—C6—C5	118.94 (15)	N3—C9—C8	119.55 (15)
C1—C6—C7	119.56 (14)	N2—C8—N1	118.36 (14)
C5—C6—C7	121.50 (14)	N2—C8—C9	123.34 (14)
C2—C1—C6	120.50 (16)	N1—C8—C9	118.29 (14)
C2—C1—H1A	121.0 (12)	C8—N1—C12	123.30 (14)
C6—C1—H1A	118.5 (12)	C8—N1—H1N1	117.7 (12)
C1—C2—C3	120.25 (17)	C12—N1—H1N1	119.0 (12)
C1—C2—H2A	120.4 (12)	C8—N2—H1N2	118.9 (12)
C3—C2—H2A	119.3 (12)	C8—N2—H2N2	118.3 (14)
C4—C3—C2	119.53 (17)	H1N2—N2—H2N2	116.3 (18)
C4—C3—H3A	121.2 (11)	C9—N3—H1N3	117.9 (13)
C2—C3—H3A	119.3 (11)	C9—N3—H2N3	114.0 (12)
C3—C4—C5	120.59 (17)	H1N3—N3—H2N3	118.1 (17)
C3—C4—H4A	120.4 (13)	C11—C12—N1	119.87 (15)
C5—C4—H4A	119.0 (13)	C11—C12—H12A	125.2 (11)
C4—C5—C6	120.18 (17)	N1—C12—H12A	114.9 (11)
C4—C5—H5A	119.9 (11)	C12—C11—C10	118.99 (16)
C6—C5—H5A	119.9 (11)	C12—C11—H11A	122.1 (11)
O1—C7—O2	123.33 (14)	C10—C11—H11A	118.9 (11)
O1—C7—C6	118.52 (14)	C9—C10—C11	121.30 (16)
O2—C7—C6	118.15 (13)	C9—C10—H10A	119.5 (10)
C10—C9—N3	122.21 (15)	C11—C10—H10A	119.2 (10)
C10—C9—C8	118.22 (14)		
			
C5—C6—C1—C2	0.5 (2)	C10—C9—C8—N2	−179.52 (15)
C7—C6—C1—C2	−179.77 (15)	N3—C9—C8—N2	1.9 (2)
C6—C1—C2—C3	−0.3 (3)	C10—C9—C8—N1	1.4 (2)
C1—C2—C3—C4	−0.1 (3)	N3—C9—C8—N1	−177.16 (13)
C2—C3—C4—C5	0.5 (3)	N2—C8—N1—C12	−179.52 (14)
C3—C4—C5—C6	−0.3 (3)	C9—C8—N1—C12	−0.4 (2)
C1—C6—C5—C4	−0.1 (3)	C8—N1—C12—C11	−0.4 (2)
C7—C6—C5—C4	−179.90 (16)	N1—C12—C11—C10	0.2 (2)
C1—C6—C7—O1	−10.6 (2)	N3—C9—C10—C11	176.89 (15)
C5—C6—C7—O1	169.17 (15)	C8—C9—C10—C11	−1.7 (2)
C1—C6—C7—O2	169.17 (14)	C12—C11—C10—C9	0.8 (3)
C5—C6—C7—O2	−11.1 (2)		

### Hydrogen-bond geometry (Å, °)

**Table:**

*D*—H···*A*	*D*—H	H···*A*	*D*···*A*	*D*—H···*A*
N1—H1N1···O2	0.96 (2)	1.77 (2)	2.7218 (18)	176 (2)
N2—H1N2···O1	0.90 (2)	1.94 (2)	2.8377 (18)	173 (2)
N2—H2N2···O2^i^	0.88 (2)	2.01 (2)	2.8873 (17)	170 (2)
N3—H1N3···O2^i^	0.91 (2)	2.02 (2)	2.9206 (19)	173 (2)
N3—H2N3···O1^ii^	0.95 (2)	2.00 (2)	2.9382 (19)	170 (2)

Symmetry codes: (i) −*x*+1/2, *y*−1/2, *z*; (ii) *x*−1/2, −*y*+1/2, −*z*+1.

## References

[bb1] Allen, F. H., Kennard, O., Watson, D. G., Brammer, L., Orpen, A. G. & Taylor, R. (1987). *J. Chem. Soc. Perkin Trans. 2*, pp. S1–19.

[bb2] Bernstein, J., Davis, R. E., Shimoni, L. & Chang, N.-L. (1995). *Angew. Chem. Int. Ed. Engl.* **34**, 1555–1573.

[bb3] Bruker (2005). *APEX2*, *SAINT* and *SADABS* Bruker AXS Inc., Madison, Wisconsin, USA.

[bb4] Cosier, J. & Glazer, A. M. (1986). *J. Appl. Cryst.* **19**, 105–107.

[bb5] Jeffrey, G. A. (1997). In *An Introduction to Hydrogen Bonding* Oxford University Press.

[bb6] Jeffrey, G. A. & Saenger, W. (1991). In *Hydrogen Bonding in Biological Structures* Berlin: Springer.

[bb7] Katritzky, A. R., Rees, C. W. & Scriven, E. F. V. (1996). In *Comprehensive Heterocyclic Chemistry II* Oxford: Pergamon Press.

[bb8] Pozharski, A. F., Soldatenkov, A. T. & Katritzky, A. R. (1997). In *Heterocycles in Life and Society* New York: Wiley.

[bb9] Scheiner, S. (1997). In *Hydrogen Bonding, A Theoretical Perspective* Oxford University Press.

[bb10] Sheldrick, G. M. (2008). *Acta Cryst.* A**64**, 112–122. 10.1107/S0108767307043930 18156677

[bb11] Spek, A. L. (2009). *Acta Cryst.* D**65**, 148–155. 10.1107/S090744490804362X PMC2631630 19171970

